# Supplementary figures and images for: Extant life detection using label-free video microscopy in analog aquatic environments
Source: PLoS One. 2025 Mar 12;20(3):e0318239. doi: 10.1371/journal.pone.0318239 (PMC11902266; doi:10.1371/journal.pone.0318239)

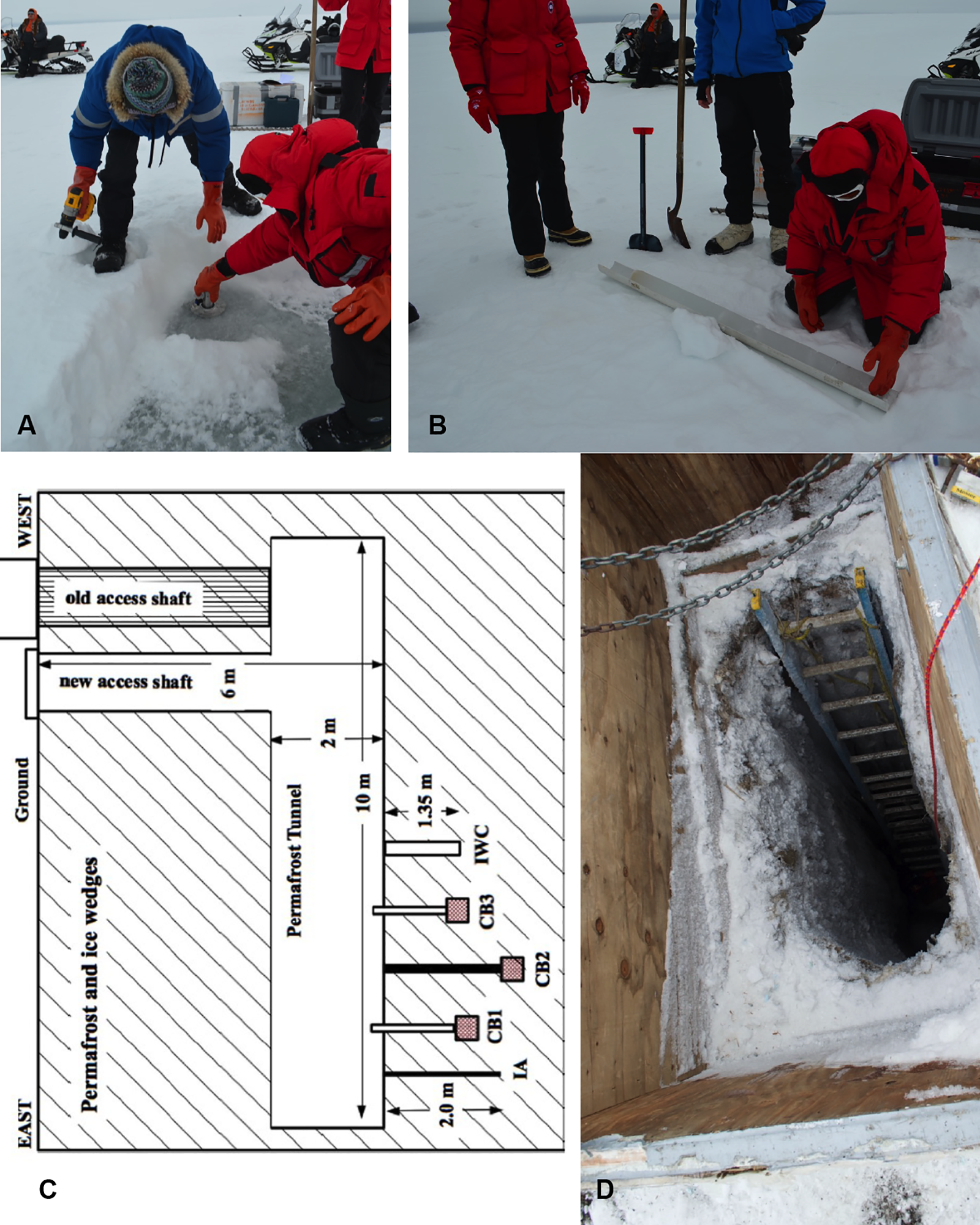

Supplement: S1 Fig — (A) Removing snow to access sea ice for coring and brine sampling. (B) Sea ice core. (C) Diagram of tunnel allowing access to cryopegs. (D) Photo of access tunnel. (TIF) [file pone.0318239.s001.tif]

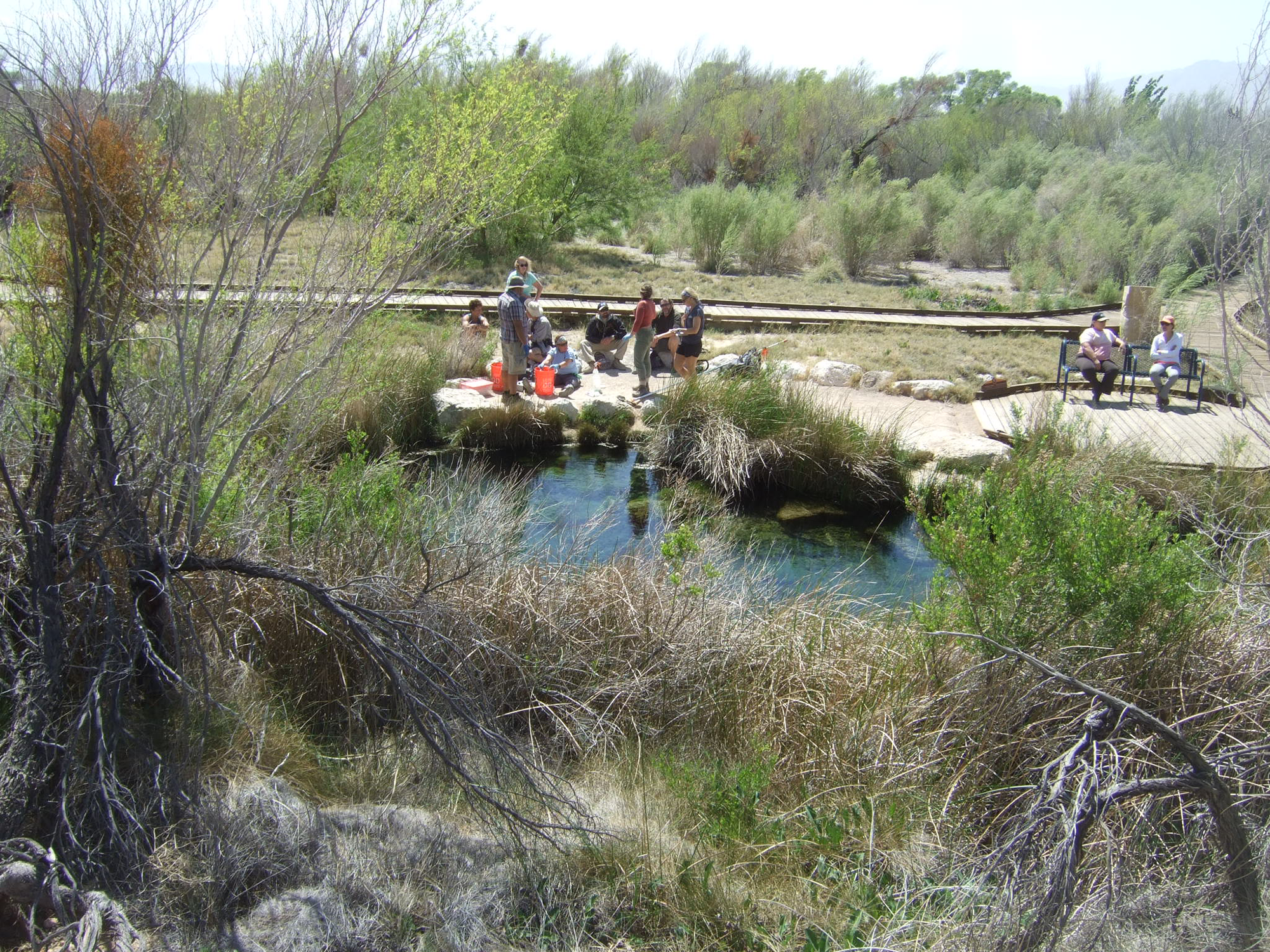

Supplement: S2 Fig — Within the boundary of Ash Meadows National Wildlife Refuge exists many spring fed pools, imaged here is Kings Pool. (TIF) [file pone.0318239.s002.tif]

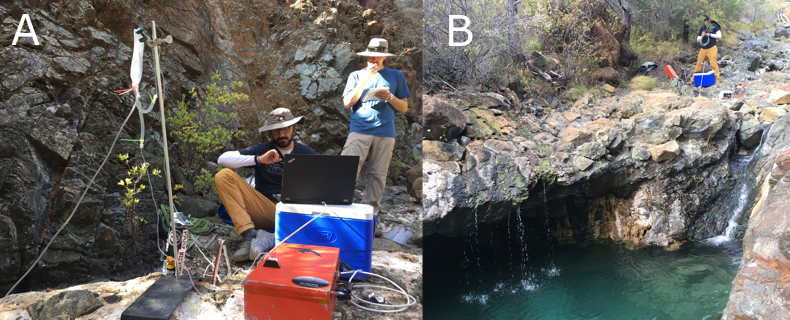

Supplement: S3 Fig — (A) Shows the collecting of DHM data from BS5 using the SHAMU instrument. (B) A photo of GPS and Manuel Bedrossian preparing tubing for sample collection. (TIF) [file pone.0318239.s003.tif]

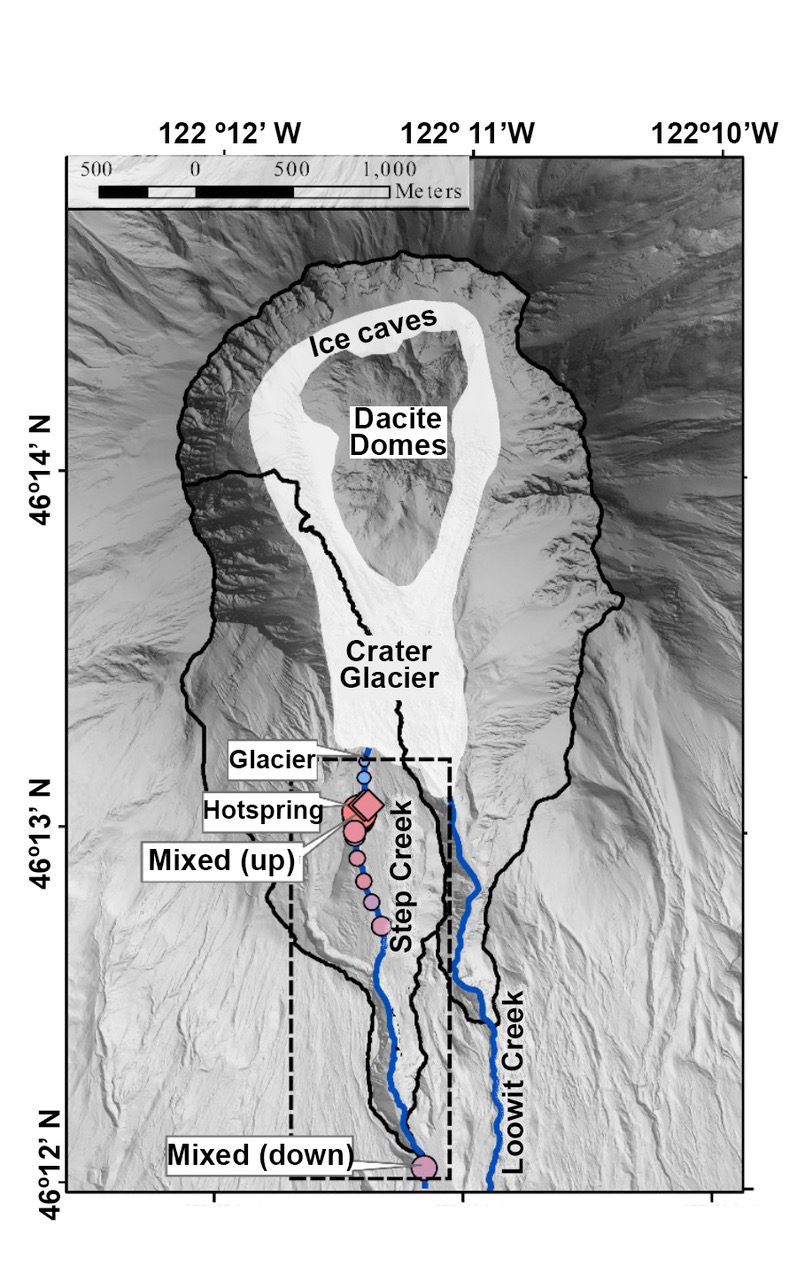

Supplement: S4 Fig — The sites reported here are glacier discharge (upstream), the site of mixing of glacier discharge with hot spring (Midstream or Mixed (up)), and mixed downstream. Basemap derived from USGS Data Series 904 authors by Adam Mosbrucker [103]. (TIF) [file pone.0318239.s004.tif]

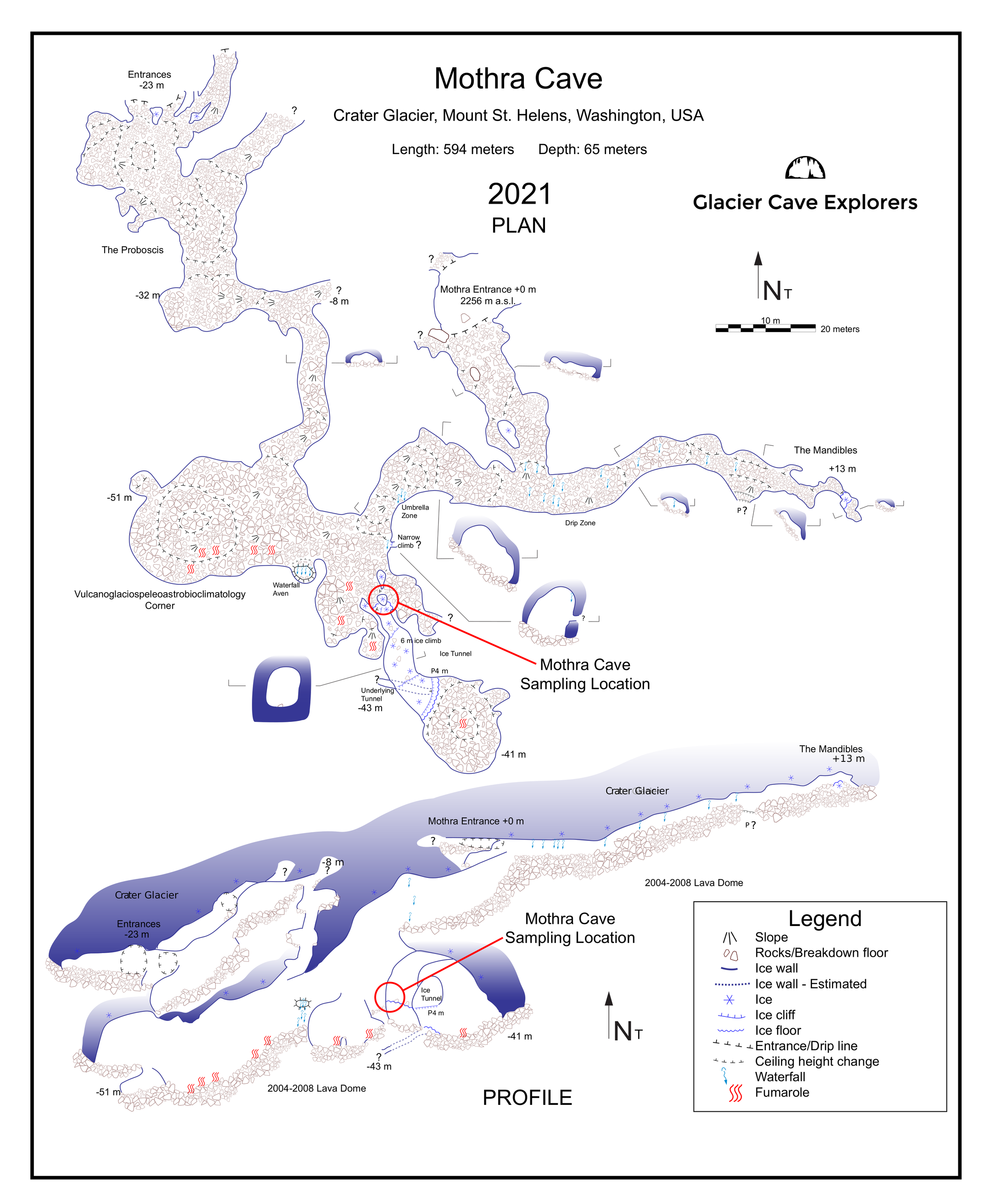

Supplement: S5 Fig — This map was derived during the fieldwork within the Mothra cave. The top depiction of the glaciovolcanic cave is from above looking down towards the center of the Earth. The bottom depiction shows the profile or cross section of the cave. The orientation of the profile image corresponds to the top depiction of the cave. Republished from [40], Image by Christian Stenner licensed under CC-BY 4.0. (TIF) [file pone.0318239.s005.tif]

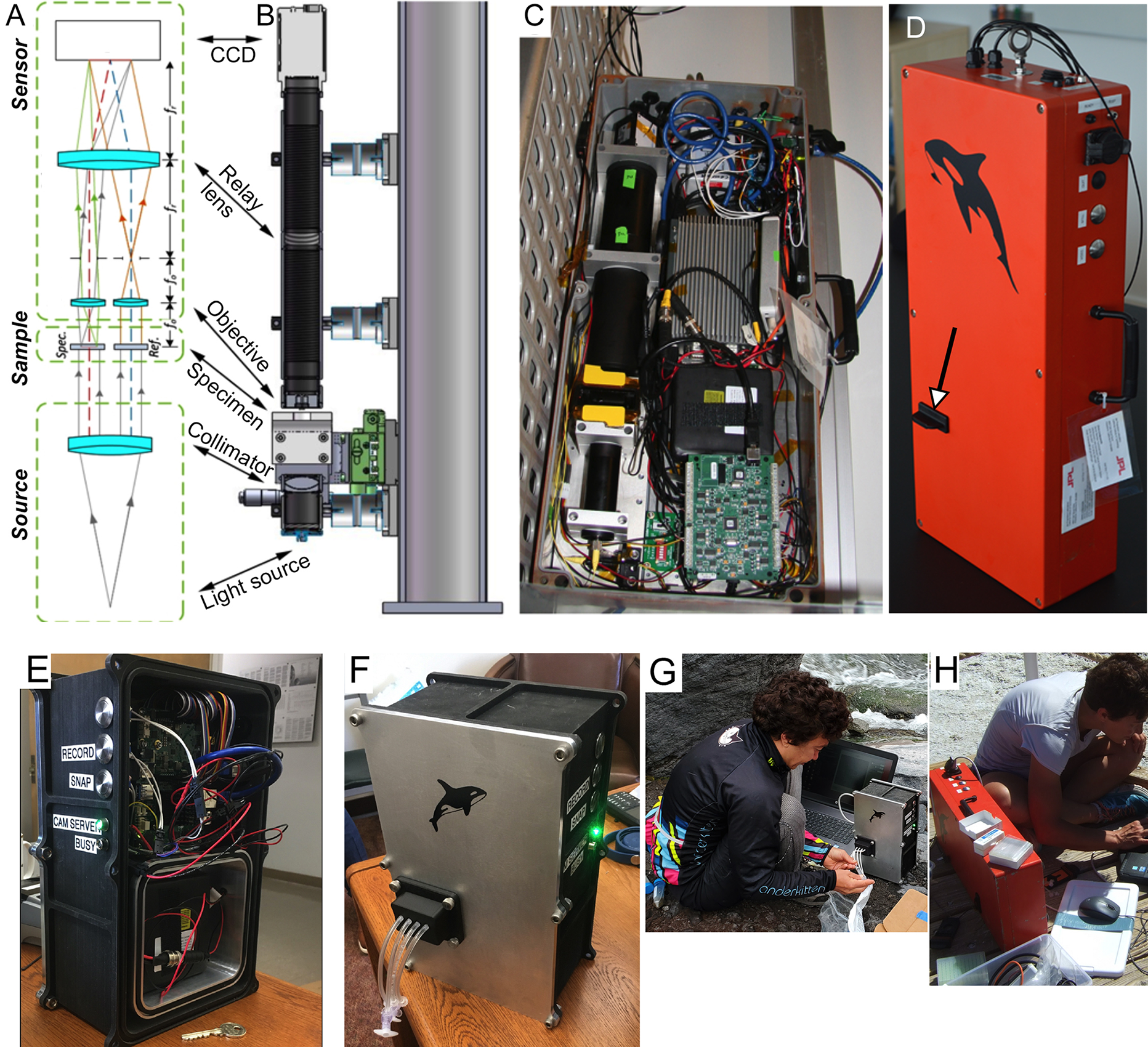

Supplement: S6 Fig — (A, B) Optical train common to both designs. (C, D) Original field design, called SHAMU, total weight 10 kg (base images A-D previously published in [27]). (E, F) Compact folded field design, Son of SHAMU, total weight 6 kg. (G) Son of SHAMU in the Mt. St. Helens crater. (H) SHAMU in Death Valley. (TIF) [file pone.0318239.s006.tif]

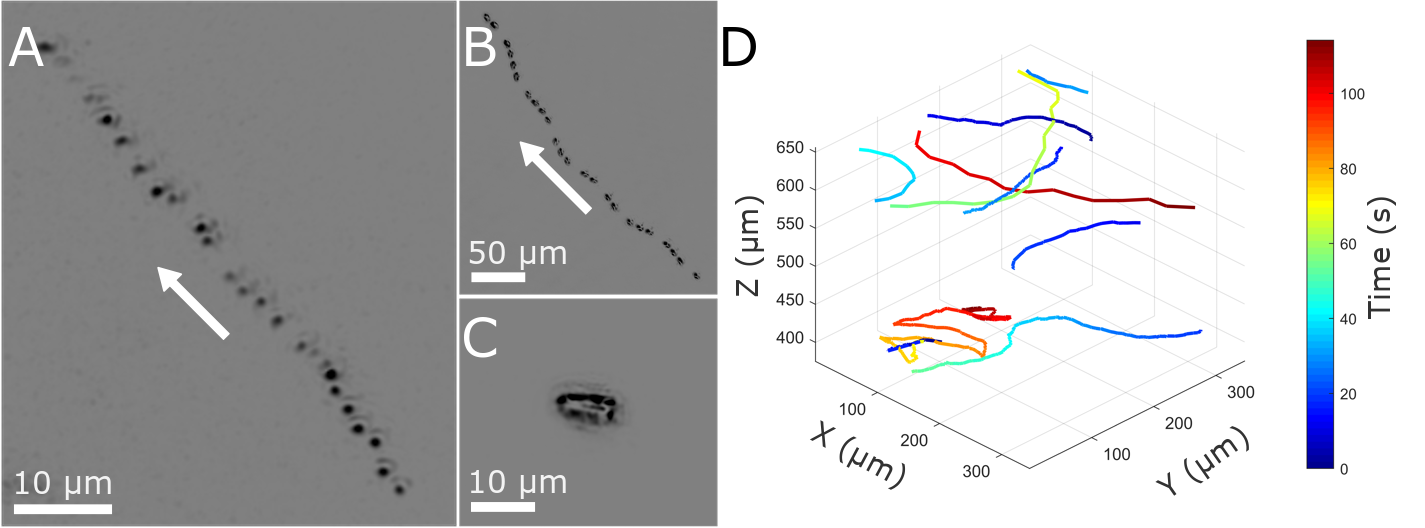

Supplement: S7 Fig — (A) Select trajectory of a motile microorganism found in the sea ice brine sample. This image is a composite image rendered by the minimum pixel intensity projection of 5 seconds of data. (B) Select trajectory of a motile microorganism found in the ice/ocean interface sample. This image is a composite image rendered by the minimum pixel intensity projection of 5 seconds of data. (C) A single plane intensity reconstruction showing a select microorganism observed at the ice/ocean interface sample. (D) Trajectories of the heterogeneous in situ sample taken from the ice/ocean interface. This plot shows trajectories of multiple organisms within the field of view of the DHM instrument and is color coded with respect to time. (TIF) [file pone.0318239.s007.tif]

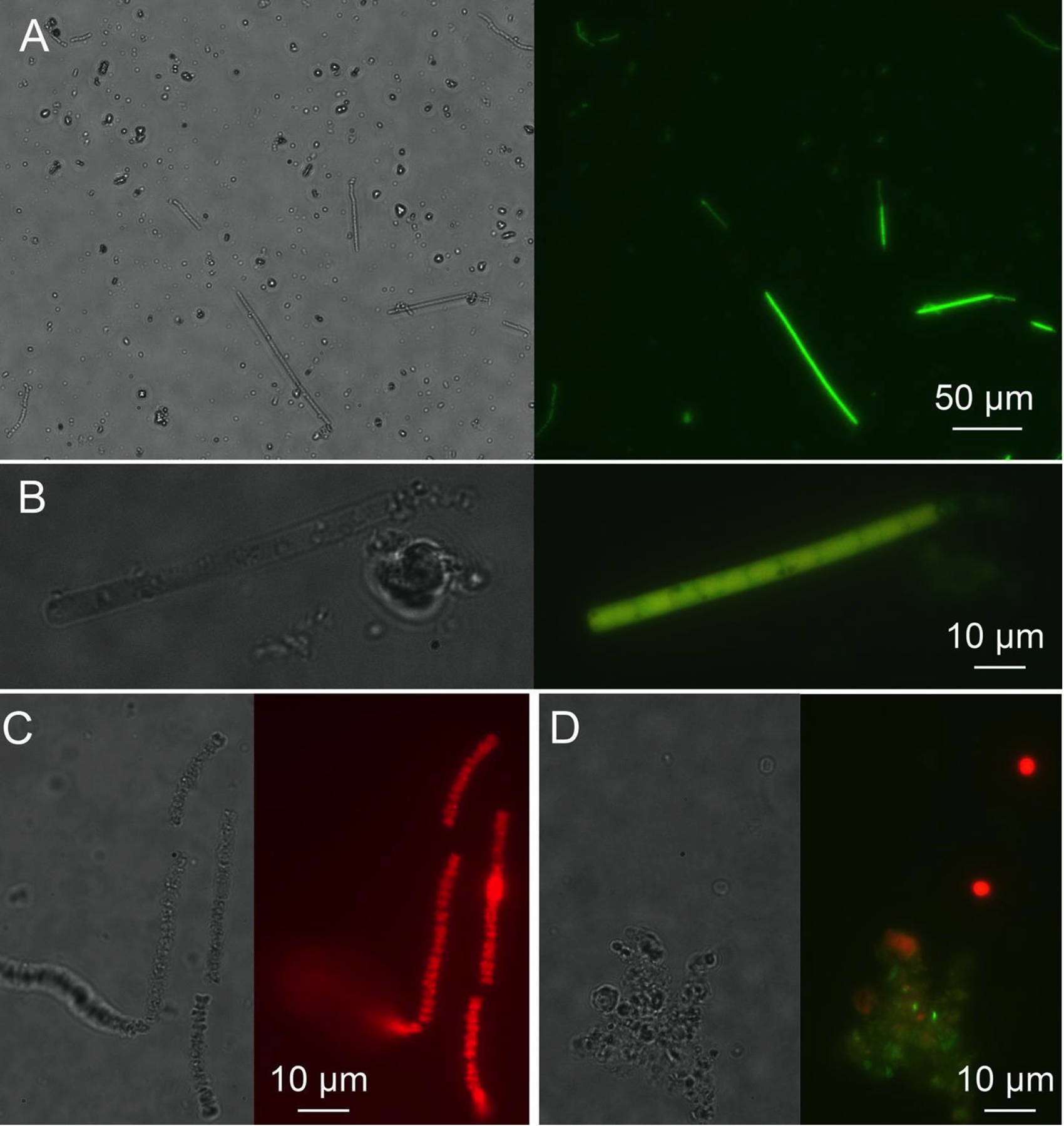

Supplement: S8 Fig — (A) Low-power image of Salton Sea sample stained with SYTO9, under phase contrast (left) and fluorescence (right). (B) High-power image of a single large microbe stained with SYTO9, under phase contrast (left) and fluorescence (right). (C) Unstained Salton Sea large microbes showing autofluorescence upon green excitation, under phase contrast (left) and fluorescence (right). (D) Salton Sea sample stained with SYTO9 and demonstrating red autofluorescence, showing the fusion of the green and red channels. (TIF) [file pone.0318239.s008.tif]

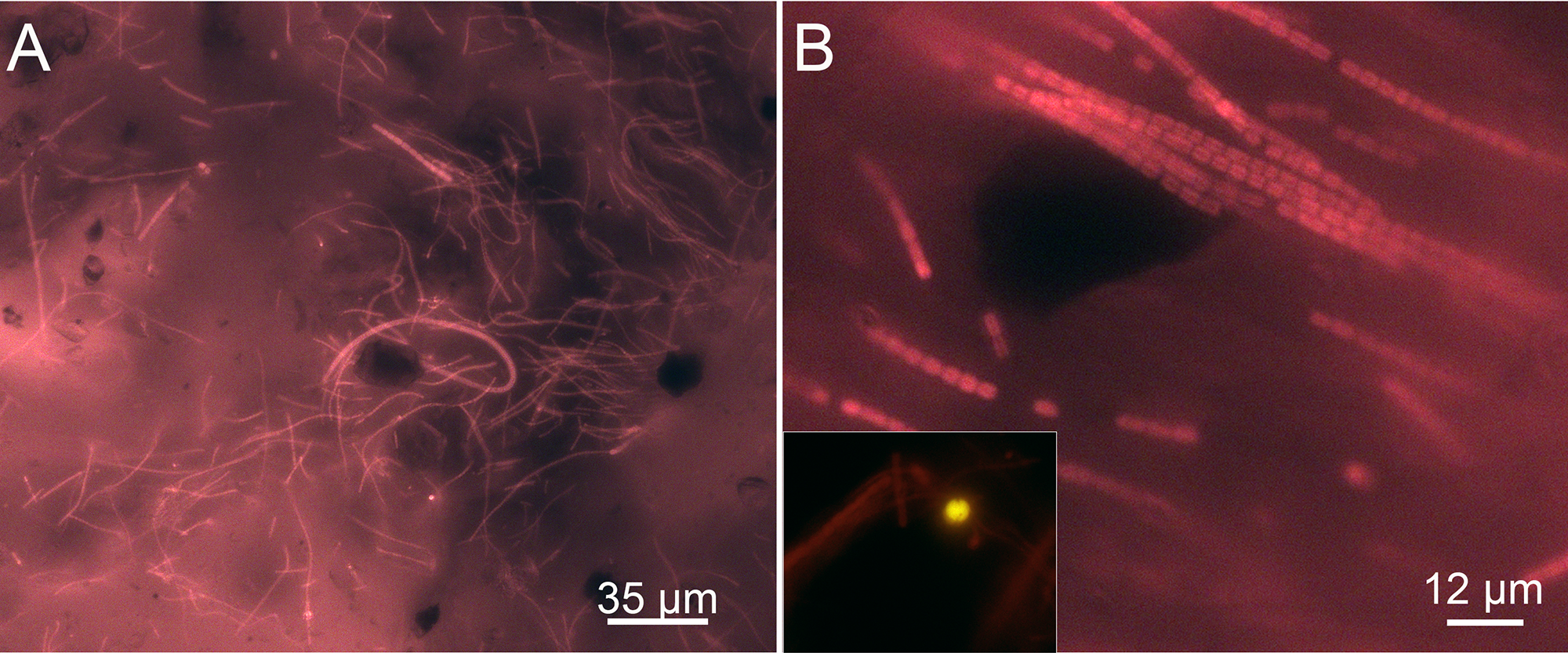

Supplement: S9 Fig — (A) Low power image of mat chlorophyll autofluorescence. The dark areas are minerals. (B) Higher power image showing segmented photosynthetic cells. The inset shows SYTO9 staining of a round photosynthetic cell. (TIF) [file pone.0318239.s009.tif]

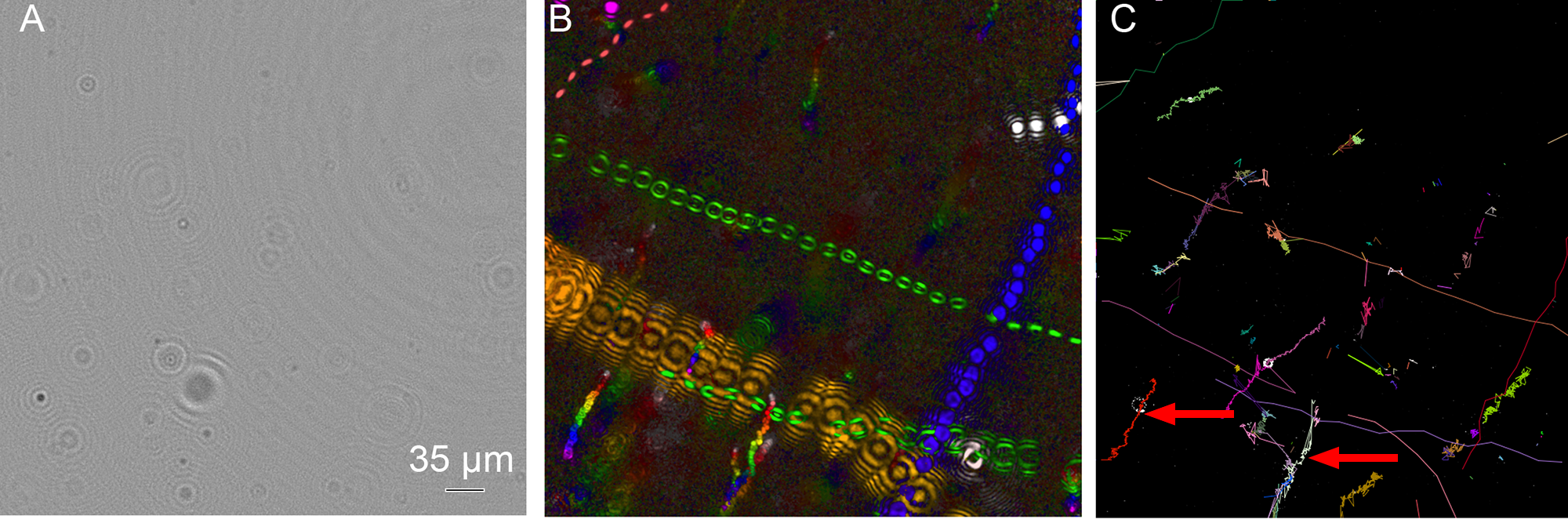

Supplement: S10 Fig — (A) Amplitude reconstruction of a single plane showing several 2-5 μm sized cells. (B) MHI of recording showing rapid swimming of 5 different cells within the 30 s time frame of the video. (C) Tracks of cells in (B). Red arrows are pointing to tracks of drifting cells that would be ideal for using to apply drift subtraction of motile cells. (TIF) [file pone.0318239.s010.tif]
