# Supplementary material for: Extant life detection using label-free video microscopy in analog aquatic environments
Source: PLoS One. 2025 Mar 12;20(3):e0318239. doi: 10.1371/journal.pone.0318239 (PMC11902266; doi:10.1371/journal.pone.0318239)
Supplement: Attachment — Submitted filename: ReviewPONE-D-23-22462.docx [file pone.0318239.s031.docx]

PONE-D-23-22462

# Summary

The authors present results using holographic microscope in situ from a collection of various environments known to be extreme for life (ice brines, hypersaline pools,etc…), based on prototypes (one of them have been previously published). The authors also present stimuli to interfere with cell motility. Such results could have some useful information for astrobiology to justify to use of holography microscopy on a mission.

However, if they provide a catalog of various results in term of biological environment, taking into account on the difficulties to get such results in situ, still, the number of sample per site is limited which makes difficult to get a statistically strong measurement.

Author provide an overview of biosignature they obtained, but unfortunately many are not quantified even when it is abundant. Such quantification, with a homogenous method and sufficient statistics would really help providing significant biosignature.

# Example and evidences

## Major issues

Some results have already published : fig 3 was present in Fig 6 in their paper (26) , Fig S6 was Fig1 for instance.

Careful relecture is necessary. For instance, 1) some figures doesn’t correspond: in Figure 3 D to F subpanels are absent. 2) in the text, mismatch between Fig S7 and S8. 3) for Badwater Basin, “motile cells” is used but not clear if it concerns eukaryotes or prokaryotes.

In some movies, we can see global drift. It is not clear how it has been taken into account

For some results, (in different sections, only 2 or 3 cells have been measured), which is really too low. Moreover, it makes difficult to consider a Standard Deviation.

## Minor issues

Using time in seconds instead of frame number would help for HMI. For the same reason, adding a timestamp in movies helps to get an idea of the timescale.

Movies contrast is variable, which makes difficult to evaluate. Having all the data with same contrast would be useful
