# Supplementary material for: Extant life detection using label-free video microscopy in analog aquatic environments
Source: PLoS One. 2025 Mar 12;20(3):e0318239. doi: 10.1371/journal.pone.0318239 (PMC11902266; doi:10.1371/journal.pone.0318239)
Supplement: Attachment — Submitted filename: Response to Reviewers.docx [file pone.0318239.s032.docx]

**PLOS ONE Manuscript PONE-D-23-22462**

**Response to Reviewers**

Dear PLOS ONE editor and reviewers,

Thank you for giving us the opportunity to submit a revised draft of the manuscript “**Extant Life Detection Using Label-Free Video Microscopy in Analog Aquatic Environments**” for publication in the PLOS ONE Journal. We recognize and greatly appreciate the time and labor that you all dedicated to providing input on improving our manuscript and are grateful for the insightful critiques on and valuable improvements to our paper. We have included many of the suggestions made. All changes within the manuscript are seen in red text. Please see below, in blue, for a point-by-point response to the reviewers’ comments and concerns. All page numbers refer to the revised manuscript file with tracked changes.

**Reviewers' Comments to the Authors and Respective Responses to Reviewers:**

**Reviewer 1**

I recommend publishing the article in spite of the lack of sufficient statistics, detected by reviewers, based on our experience on extreme environments. I think that the authors have adequately proposed the statistics that can be done in such kind of environments, which is valuable for going ahead.

**Author response**: Thank you for your comments and approval for publication.

**Reviewer 2**

Thanks for the author to the important effort to improve their manuscript which is clearly easier to read.
Based on their feedback, I’m still confused about the take-home message and how they provided the sufficiently argued elements.

Again, I really appreciate the effort of the authors to improve the text, but several weaknesses in the data analysis seem to me too important to publish under these conditions.

**Author response**: Thank you for your kind words as well as taking time to review and suggest further edits. These comments help us strengthen our paper and make it easier for future readers to understand our work.

During this round of edits, we further tried to improve our paper by implementing the following:

- Solidifying that the main purpose of the paper is to demonstrate the presence and ability of the DHM to detect active motion, morphology, and optical property biosignatures for a variety of environments.
- Adding the argument that limited data sets and ground truthing are similar to what we would expect from off-world experiments. Therefore, knowing what claims can be made with the limited data we have in this paper is useful for future off-world astrobiology missions.
- Adding ground truthing for all sites, except one.
- Removing characterization of swimming speeds, which adds to the consistency of the quantitative results.
- Including displacement comparison of Brownian motion to suspected motility tracks.
- Including accuracy metrics as validation for automated HELM method.
- Adding the argument for manual tracking and classifying motion as either biotic or abiotic as gold standard for accuracy.

The main take-home message is that active motion, morphology, and optical property biosignatures are detectable from a variety of sites on Earth (mostly comprising of extreme environments that can act as analogs for ocean worlds) using DHM as the imaging technique. We have added text to the introduction, material and methods, and discussion section to make clear that the statement above is the take-home message. In the material methods section, we have added subsections outlining how active motion, morphology, and optical properties are biosignatures and how they were identified. Below is the text that was added to the paper regarding this point.

**INTRODUCTION:**

L57 –

Techniques for identifying these features of life (i.e. biosignatures) are central to astrobiological goals of detecting and possibly studying life on other planetary bodies. This study investigates the ability of digital holographic microscopy to conduct *in situ* experiments to detect and classify the prevalence of active motion, morphology, and optical properties of microbial cells as biosignatures of extant life across a variety of extreme environments here on Earth.

L70 –

Active motion in this paper, as detailed in the methods section is characterized as any cell-like object that is moving in a way that is distinct from any known abiotic motion (i.e. Brownian, fluid flow, buoyancy) [5, 7].

With non-motile cells, simple visualization of morphologically complex organisms in natural samples can often be sufficient to conclude that they are alive [5, 8-10]. With smaller organisms —bacteria and archaea—the situation is more complicated. Their cells contain few features to suggest metabolism or complexity, but their optical properties, refractive index and absorbance, alter their contrast uniquely relative to most microminerals present in *in situ* samples [11]. Therefore, non-motile organisms can be classified by their distinct appearance due to their morphological features or optical properties relative to abiotic objects.

L99 –

The purpose of collecting *in situ* data from a variety of extreme environments and stimulation experiments here on Earth is to start to build a framework for detecting biosignatures with a DHM instrument for possible future extant life detection missions on a range of planetary body analog environments. All three microbial biosignatures we examined (i.e. active motion, morphology, and optical properties) are present in almost all sites and at least one is present in every site. The hypothesis of this work is that while not every microbe will exhibit detectable motion, morphology, or optical property biosignatures, within most environments – no matter how extreme – some fraction of microbes will be identifiable by these biosignatures. The fraction of motile cells may be increased by stimulation with heat [17], simple nutrients [18], salinity gradients [19], or other chemical or physical attractants or repellents [20. 21].

**MATERIAL AND METHODS:**

## L255 –

## 4. Biosignatures

Trained experts manually identified and classified active motion, morphology, and optical properties as biosignatures through inspection of x, y, t stacks; x, y images; MHIs; and plotted tracks of particles. False positive rates for manual identification of biosignatures, while theoretically not zero, can be considered negligible [53, 54].

### 4.1 Active vs. passive motion

Active motion is characterized by distinguishing the motion from known forms of passive motion (e.g. Brownian, fluid flow, buoyancy) [7. 61]. Fluid flow is readily characterized as unidirectional motion of all particles in the field of view, and may be subtracted readily by computer either separately or in conjunction with track analysis. HELM, as well as other particle trackers such as MOSAIC or NTA NanoTrackJ [64], distinguish Brownian motion by plotting root mean squared displacement *d* vs. time for each tracked particle. Brownian motion scales with the square root of time t:

$d= \sqrt{2Dt}$	(1)

where D is the diffusion coefficient given as,

$D={kT}/{6\pi\eta r}$	(2).

In (2) *k* is the Boltzmann constant, *T* is temperature, $\eta$ is the viscosity of the fluid, and *r* is the radius of the particle. Manual distinguishing of Brownian vs. active motion is based upon observation of displacement and is nearly always unambiguous as shown in **Fig. 4**. At room temperature (25ºC) with a 1 µm radius particle in water, the diffusion coefficient is ~0.24 µm^2^/s. At 4ºC the viscosity of water is substantially greater, giving a diffusion coefficient of ~0.13 µm^2^/s. Measured bacterial diffusion coefficients can be 1000-2000 times greater than predicted when cells aggregate [65]. Making it important to ensure that aggregates be distinguished from single cells by imaging, tracking, or both. Rapidly swimming cells (v = 100 µm/s) displace much more rapidly than Brownian particles (**Fig. 4A**). Even moderate swimming speeds of 10 µm/s are readily distinguished from Brownian motion of single cells; aggregates can be identified by displacement vs. time curves or simply eliminated from manual tracks (**Fig. 4B**). More slowly swimming cells are distinguished by their displacement vs. time curves (**Fig. 4C**). We have previously studied Brownian motion of microparticles and non-motile cells in depth and found that displacements match the models well and that displacement vs. time curves can be used to identify passive diffusion [7].

Along with directional drift and displacement-vs.-time curves, HELM also uses other track parameters such as sinuosity and angle of displacement to identify motile particles. In lab monoculture samples, the true positive rate over the false positive rate for automated motile track detection was reported to be 0.92 [61]. Manual results remain superior to any automated results, however, largely due to poor stitching of automatic tracks. Manual tracking combined with plotting displacement curves in the case of slow-moving cells essentially eliminates false positive identification of motile tracks.

The number of observed motile organisms in our samples was highly variable; some sites had only a few organisms identified over many recordings while some had dozens per field of view. This variability led us to use a qualitative analytical approach where motile organisms were classified as either highly abundant (multiple motile cells in each frame), abundant (at least one motile cell per frame), present (motile cells in some but not all frames), or not present (no motile cells in any frame).

**Figure 4. Brownian motion vs swimming.** (A) Fast swimming, 100 µm/s, is clearly differentiated from Brownian motion even at short time scales of recording. (B) Moderate swimming speeds of 10 µm/s are still many times faster than Brownian motion of single cells and can be differentiated from Brownian motion of aggregates by displacement vs. time curves. (C) The slower swimming and diffusion rates seen at low temperatures can still be readily distinguished, although longer recordings are helpful to capture the shape of the curves.

### 4.2 Morphology

Morphological features can demonstrate a complexity that is an unambiguous biosignature. Morphology features include organelles, filamentous, appendages, and cellular clustering [5, 9, 10]. The resolution limit of our DHM instrument allows for the identification of organelles in larger eukaryotes. Cells identified to have organelles were classified as “large” cells and were generally >20 µm in diameter. “Small” cells were those <5 µm in diameter where no organelles could be distinguished, and cells could not be classified as eukaryotic or prokaryotic by DHM alone, as the environments studied often contained an abundance of small eukaryotes [66,67].

### 4.3 Optical properties

Absorbance and refractive index can both be used as biosignatures as both amplitude and phase images allow one to distinguish cells from microminerals. The difference in absorbance and refractive index of minerals, relative to cells, is generally represented in our microscopy images as higher contrast and sharper edges. Trained scientists can use these features alongside morphology and motion to label a particle as likely a cell or not.

Contrast in amplitude images is given by a combination of absorbance and scattering:

$\sigma=\sigma_{abs}+\sigma_{scatt}$	(3)

For absorbing particles, the absorbance term is approximately equal to the absorbance *A* as calculated from the Beer-Lambert Law,

$A=\varepsilon\left( \lambda\right)ct$	(4)

where $\varepsilon\left( \lambda\right)$ is the extinction coefficient (or imaginary part of the refractive index, a measure of how much light is being absorbed at a given wavelength) of the absorbing molecule, *c* is its concentration, and *t* is the thickness of the cell. With our illumination wavelength at 405 nm, chlorophyll is strongly absorbing, so that photosynthetic cells appeared dark. Many minerals, in contrast, are reflective rather than absorbing and appeared bright in amplitude. Small, nearly transparent cells such as bacteria are dominated by the scattering term, which can be approximated using Mie scattering theory or calculated more accurately for non-spherical cells using the discrete dipole approximation.

In phase reconstructions from DHM images, contrast $\Delta\phi$is given by

$\Delta\phi=2\pi t\Delta n/\lambda$	(5)

where $\lambda$ is the wavelength of illumination, *t* is the thickness of the specimen, and $\Delta n$ is the difference in (imaginary) refractive index between the object and its surrounding medium (in this case water, with *n* = 1.33). Most cells have $\Delta n\sim0.05$, whereas minerals have values 5-10 fold higher [11]. The limit of detection with a single exposure in our system was approximately $\frac{\Delta\phi}{2\pi}=0.05$, or $\frac{t}{\lambda}\sim1$ for detection of cells.

L656 –

# Discussion

As indicated in a 2016 NASA report, microscope-based identification of cell-like objects on future missions to other planetary bodies, whether or not the objects are definitively alive, will assist other means of characterization such as mass spectrometry, Raman or infrared spectroscopy, or other chemical analyses [3]. Chemical signals coming from a known self-contained object, or a selection of such objects, will be more compelling as biosignatures when images showing the objects accompany the chemical data contain biosignatures of their own. This study gives context on how to best use a DHM instrument to detect biosignatures in extreme environments. The *in situ* identification of at least one biosignature (i.e. motion, morphology, or optical properties) was confirmed at every site. Even though not all biosignatures were present in each recording, these results help build a case and framework for how to search for these biosignatures during future extant life detection missions.

Label-free amplitude and phase video microscopy can provide an array of biosignatures, some definitive and others suggestive. Either active motility or clear morphological features, if observed on another planet, would be a compelling biosignature, especially in combination with chemical studies demonstrating the presence of complex molecules and/or disequilibrium. Absorbance and refractive index optical properties are not definitive biosignatures on their own but fall into this suggestive category. The prevalence of motility, morphology, and optical property biosignatures in analog sites of astrobiological significance has remained largely unexplored. In this study, we observed motility and optical property biosignatures in samples from all the environments tested. Morphological features were identified in all environments except for cryopeg, alkaline spring pool, and subglacial void. The slowest microbes moved well above distinguishable speeds from Brownian and other forms of abiotic motion. Simple stimuli resulted in dramatic increases in motility in seawater and sea ice brines. Raising the temperature of sea ice brines from ambient (< 0ºC) to 4ºC resulted in small microbial motility, as did addition of 1 mM L-serine (but not other amino acids or glucose). Gradients of L-serine were not required to observe motility, though they could be used for further confirmation of directed swimming. Larger, clearly eukaryotic organisms were spontaneously motile in all sea ice and brine samples.

Further descriptions of how we implemented the other improvements are detailed in the responses below.

1. If I fully understand that the measures on the different sites were done by different teams having their own major goals. However, the variability of the results (image quality , manual/automatic tracking, etc…) could be due to sites or experimentalist. My understanding of providing a biosignature implies a robust and reproducible protocol. For instance L301 : The x, y, t stacks used were usually z **projections over multiple planes, but occasionally better SNR was obtained using a single denoised z-**plane reconstruction rather than a projection. In Table 2 for the same concentration category, speed is quantified or not among the different sites. In the same idea, claiming an autonomous software package is slightly overselling as authors explain to change of analysis pipeline depending on the SNR of the images.

**Author response**: Yes, while it is accurate to state that data collection was done by different teams with their own major goals, it is important to note that image processing and biosignature detection was done in similar manner for each site. By similar we mean that we first attempted to clean up data (as described in paper) and then identified, tracked, and classified particles as motile or not using automated methods. We then confirmed manually these tracks and classifications. If there were too many false positives or false negatives using the automated method then we moved to manually identifying, tracking, and classifying cells. Manual particle tracking and classification of motility is considered to be the most accurate method.

To make the workflow clearer for the reader we revised the image processing section and added a biosignature section. Below is the text for the image processing section, as the biosignature section is listed above.

L193 –

## Image processing

The aim of image processing was to improve the signal-to-noise ratio (SNR) of the particles to make them easier to identify, characterize as cell-like (or not), and track. A general image processing protocol can be seen in **Fig. 3**. Until the last few decades all microscopy-based cellular classification and tracking was done manually by trained researchers [51]. Now that computers have become capable of single particle tracking and classifying motion, many more particles may be tracked at a time in some datasets, but manual tracking remains the most accurate method for discriminating particles from noise and linking tracks of a single particle [52-57]. No one approach works for all datasets, especially when sizes and speeds of particles vary and when samples represent a heterogeneous mix of particle types and noise. Thus, the process of reducing noise, localizing particles, and linking trajectories for our *in situ* experiments was unique for each recording, iterative, and a combination of manual and automated methods. We detail this carefully here in order to inform future similar work, as data processing techniques and details of manual tracking are often poorly described in the literature or tracking techniques are illustrated only for homogeneous populations with known particle shape and size.

L218 –

The next step in the analysis pipeline was to process the x, y, t stacks with HELM, a software designed to characterize motility autonomously, using realistic flight mission computational resources, that has been characterized previously in detail [61-63]. The choice of amplitude or phase images for use in tracking also depended upon the recording. HELM identifies pixel changes in sequential DHM images, tracks clusters of change as particle movement, and classifies particles as motile or non-motile based on their movement patterns. These patterns are analyzed and classified by characterizing 23 track features (e.g. Brownian motion, fluid flow, etc.), and then passing these features through a machine learning model [61]. A motion history image (MHI) summarizes a full video in one image by color mapping each pixel to the time index of largest intensity change; this image was used to quickly analyze whether motility was present in a sample and to help guide manual tracking. The x, y, t stacks used were usually z-projections over multiple planes, but occasionally better SNR was obtained using a single de-noised z-plane reconstruction rather than a projection. A researcher manually reviewed the HELM classifications to confirm accuracy and alongside this review process identified morphology and optical property biosignatures.

In datasets where false positive and false negative identifications by HELM’s classifier were never below several dozen, moving particles were instead tracked using the Fiji plugin TrackMate, using LoG/DoG detection followed by Kalman or LAP tracking [53], or Manual Tracking.

In regards to your concern that we sometimes used z-projection images or single z-plane images depending on data set to data set, we would like to relieve you of this concern. There is always going to be variability in natural samples collected *in situ,* and this variability will cause some changes in how images need to be processed. We thus described to the readers that this variability does occur and needs to be considered if trying to reproduce our results.

You brought up the fact that we quantified speed in some sites but not others in Table 2. To clean up this inconsistency we removed the speed metric from Table 2 altogether. Now speed is used only to highlight how the slowest moving cells are still notably distinguishable from Brownian motion. Below is text that highlights this transition.

L264 –

HELM, as well as other particle trackers such as MOSAIC or NTA NanoTrackJ [64], distinguish Brownian motion by plotting root mean squared displacement *d* vs. time for each tracked particle. Brownian motion scales with the square root of time t:

$d= \sqrt{2Dt}$	(1)

where D is the diffusion coefficient given as,

$D={kT}/{6\pi\eta r}$	(2).

In (2) *k* is the Boltzmann constant, *T* is temperature, $\eta$ is the viscosity of the fluid, and *r* is the radius of the particle. Manual distinguishing of Brownian vs. active motion is based upon observation of displacement and is nearly always unambiguous as shown in **Fig. 4**…

… Rapidly swimming cells (v = 100 µm/s) displace much more rapidly than Brownian particles (**Fig. 4A**). Even moderate swimming speeds of 10 µm/s are readily distinguished from Brownian motion of single cells; aggregates can be identified by displacement vs. time curves or simply eliminated from manual tracks (**Fig. 4B**). More slowly swimming cells are distinguished by their displacement vs. time curves (**Fig. 4C**). We have previously studied Brownian motion of microparticles and non-motile cells in depth and found that displacements match the models well and that displacement vs. time curves can be used to identify passive diffusion [7].

L572 –

The mean speed [± SD] of the motile microbes from this recording was 8 [± 2] µm/s, N = 6. The slowest of these motile cell moved with an average speed of over 4 µm/s, making its motility still distinguishable from Brownian motion.

L629 –

All of the tracked motile organisms were slow moving with an average speed of 6 µm/s, N = 3 (5 µm/s, 7 µm/s, 7 µm/s). Even at these speeds their motion was easily distinguished from the displacement that is caused by Brownian motion.

2. In the same idea, claiming an autonomous software package is slightly overselling as authors explain to change of analysis pipeline depending on the SNR of the images.

**Author response:** Both autonomous tracking methods, HELM and TrackMate, have been published in peer review journals where they are characterized in detail and described as autonomous software packages (referenced in the paper as [53, 61-63]). While these tracking methods are autonomous, we agree that our analysis pipeline implemented them in a user-assisted manner. We have added text that we believe makes this distinction clear to the reader in the Image Processing section of the paper as outlined below.

L218 – The next step in the analysis pipeline was to process the x, y, t stacks with HELM, a software designed to characterize motility autonomously, using realistic flight mission computational resources, that has been characterized previously in detail [61-63]. The choice of amplitude or phase images for use in tracking also depended upon the recording. HELM identifies pixel changes in sequential DHM images, tracks clusters of change as particle movement, and classifies particles as motile or non-motile based on their movement patterns. These patterns are analyzed and classified by characterizing 23 track features (e.g. Brownian motion, fluid flow, etc.), and then passing these features through a machine learning model [61]. A motion history image (MHI) summarizes a full video in one image by color mapping each pixel to the time index of largest intensity change; this image was used to quickly analyze whether motility was present in a sample and to help guide manual tracking. The x, y, t stacks used were usually z-projections over multiple planes, but occasionally better SNR was obtained using a single de-noised z-plane reconstruction rather than a projection. A researcher manually reviewed the HELM classifications to confirm accuracy, and alongside this review process identified morphology and optical property biosignatures.

In datasets where false positive and false negative identifications by HELM’s classifier were never below several dozen, moving particles were instead tracked using the Fiji plugin TrackMate, using LoG/DoG detection followed by Kalman or LAP tracking [53], or Manual Tracking. When drift was present, global drift was accounted for by tracking 3 non-motile particles (as indicated by the red arrows in **S10 Fig**) and subtracting their average instantaneous x, y velocities from the instantaneous corresponding x, y velocities of each motile organism (for more information regarding speed calculation and drift correction see **Supporting information**).

3. Active motion is presented as a key element. However, L311 it is mentioned Brownian motion analysis but we don’t understand 1) where are used / shown the results, 2) how they split between directional vs Brownian movement (as for instance NanoTrackJ is done exclusively for diffusive objects).

**Author response**: Brownian motion is a statistical phenomenon that can be described by the root mean squared displacement. The main factors at play affecting the Brownian motion in our samples are the temperature and the radius of particles. Given the range of sample temperatures and particle sizes, Brownian motion generally is distinguishable even from slow swimming cells (e.g. ~1 um/s) that are a micron in size after just 20-30 s of video. Automated methods used the root-mean squared displacement slope of the particles to distinguish Brownian motion from other forms of motion for each track created. For manual classification of cells, motion of objects were characterized as biotic or abiotic by trained experts. We feel confident in the manual process because with in the disciplines of *in situ* microbial microscopy and Single Particle Tracking (SPT) manual tracking is considered the most accurate at identifying particles and distinguishing abiotic motion from cellular swimming [1-6]. We have added a figure and the following text within the Material and Methods section.

### L260 –

### 4.1 Active vs. passive motion

Active motion is characterized by distinguishing the motion from known forms of passive motion (e.g. Brownian, fluid flow, buoyancy) [7. 61]. Fluid flow is readily characterized as unidirectional motion of all particles in the field of view, and may be subtracted readily by computer either separately or in conjunction with track analysis. HELM, as well as other particle trackers such as MOSAIC or NTA NanoTrackJ [64], distinguish Brownian motion by plotting root mean squared displacement *d* vs. time for each tracked particle. Brownian motion scales with the square root of time t:

$d= \sqrt{2Dt}$	(1)

where D is the diffusion coefficient given as,

$D={kT}/{6\pi\eta r}$	(2).

In (2) *k* is the Boltzmann constant, *T* is temperature, $\eta$ is the viscosity of the fluid, and *r* is the radius of the particle. Manual distinguishing of Brownian vs. active motion is based upon observation of displacement and is nearly always unambiguous as shown in **Fig. 4**. At room temperature (25ºC) with a 1-µm radius particle in water, the diffusion coefficient is ~0.24 µm^2^ /s. At 4ºC the viscosity of water is substantially greater, giving a diffusion coefficient of ~0.13 µm^2^/s. Measured bacterial diffusion coefficients can be 1000-2000 times greater than predicted when cells aggregate [65]. Making it important to ensure that aggregates be distinguished from single cells by imaging, tracking, or both Rapidly swimming cells (v = 100 µm/s) displace much more rapidly than Brownian particles (**Fig. 4A**). Even moderate swimming speeds of 10 µm/s are readily distinguished from Brownian motion of single cells; aggregates can be identified by displacement vs. time curves or simply eliminated from manual tracks (**Fig. 4B**). More slowly swimming cells are distinguished by their displacement vs. time curves (**Fig. 4C**). We have previously studied Brownian motion of microparticles and non-motile cells in depth and found that displacements match the models well and that displacement vs. time curves can be used to identify passive diffusion [7].

Along with directional drift and displacement-vs.-time curves, HELM also uses other track parameters such as sinuosity and angle of displacement to identify motile particles. In lab monoculture samples, the true positive rate over the false positive rate for automated motile track detection was reported to be 0.92 [61]. Manual results remain superior to any automated results, however, largely due to poor stitching of automatic tracks. Manual tracking combined with plotting displacement curves in the case of slow-moving cells essentially eliminates false positive identification of motile tracks.

The number of observed motile organisms in our samples was highly variable; some sites had only a few organisms identified over many recordings while some had dozens per field of view. This variability led us to use a qualitative analytical approach where motile organisms were classified as either highly abundant (multiple motile cells in each frame), abundant (at least one motile cell per frame), present (motile cells in some but not all frames), or not present (no motile cells in any frame).

**Figure 4. Brownian motion vs. swimming.** (A) Fast swimming, 100 µm/s, is clearly differentiated from Brownian motion even at short time scales of recording. (B) Moderate swimming speeds of 10 µm/s are still many times faster than Brownian motion of single cells and can be differentiated from Brownian motion of aggregates by displacement vs. time curves. (C) The slower swimming and diffusion rates seen at low temperatures can still be readily distinguished, although longer recordings are helpful to capture the shape of the curves.

4. Another question is still how authors split between eukaryotes and prokaryotes only based on their size. Some eukaryotes could be as small as 5µm (Ostreoccucus & others), so for a reader, it could induce erroneous information.

**Author response**: Thank you for pointing out that the method by which we are classifying Eukaryotes and Prokaryotes is prone to errors. We have taken this issue under advisement and characterized cells as either “large” or “small”. Large cells have organelle-like features while small cells we cannot determine any internal structure features.

**L310 –**

Cells identified to have organelles were classified as “large” cells and were generally >20 µm in diameter. “Small” cells were those <5 µm in diameter where no organelles could be distinguished, and cells could not be classified as eukaryotic or prokaryotic by DHM alone, as the environments studied often contained an abundance of small eukaryotes [66, 67]

**Reviewer 3**

I thank the authors for their revisions to the text in response to the reviewers' comments. Unfortunately, I cannot recommend the manuscript for publication because my comments about analysis and statistical significance (raised also by reviewer 2) have not been addressed. As I understand, PLOS ONE aims for a high degree of technical rigor, and in its current state the manuscript does not rigorously support its claims.

In particular, I remain concerned about the analysis approach, the very small number of samples, and the lack of ground truthing for several sites. The authors note in the revised manuscript that 'Cells were identified as either motile or non-motile via manual observation of the x, y, t stacks. The concentration of observed motile microbes was very low at some of the sites, with a total of only a few organisms identified over many recordings.' They further note that 'The parameters/thresholds used to process the images to increase their trackability are unique for each recording and are chosen through an iterative process of comparing identified localization of particles via the automated tracking algorithms to manual observations of the recordings at each image processing step.'

I do appreciate the amount of field work and analysis that went into this study, and the aims of the study are well-motivated. But these features alone are not sufficient for publication in PLOS ONE. The technical rigor must be improved

**Author response**: Thank you very much for taking time to review and suggest further improvements. During this round of revision, we tried to improve our paper further by implementing the following:

- Solidifying that the main purpose of the paper is to demonstrate the presences and ability of the DHM to detect active motion, morphology, and optical property biosignatures for a variety of environments.
- Adding the argument that limited data sets and ground truthing are similar to what would expect from off-world experiments. Therefore, knowing what claims can be made with the limited data we have in this paper is useful for future off-world astrobiology missions.
- Adding ground truthing for all sites, except one.
- Removing characterization of swimming speeds, which adds to the consistency of the quantitative results.
- Including displacement comparison of Brownian motion to suspected motility tracks
- Including accuracy metrics as validation for automated methods.
- Adding the argument for manual tracking and classifying motion as either biotic or abiotic as the gold standard for accuracy.

1. This approach has several major problems: (1) Relying on manual observation to identify motility makes the results subject to the particular observer and can introduce bias -- especially considering that the image processing parameters/thresholds were determined on the basis of manual observations. False positive identifications can easily result from such a procedure.

**Author response**: We appreciate you bringing up this concern. It led us to reassess our confidence in manual tracking and classifying motion as abiotic or biotic. After much discussion within our group and rereading many research publications on single particle tracking and motility classification, our confidence in our manual tracking process has been reaffirmed. Identifying motility manually is still the gold standard for accuracy. Our reasoning’s for this affirmation are listed below.

- Manual tracking and classification are regularly used as the primary analysis method [1-3, 7, 8].
- Most new methods of automated single particle tracking and motion classification as abiotic or biotic still need some manual intervention as the last step to improve the accuracy of the automated methods [4, 5, 9-11].
- To validate the accuracy of automated tracking and classifying methods researchers used datasets created manually by trained experts [6, 12-15].

Papers that are characterizing automated tracking methods are looking at clean lab cultured samples, often monocultures, or even simulated data. *In situ* samples out in the field, like the ones our paper examines, never give images as clean. This reality leads to recordings that have worse SNR and might not be as trackable with the autonomous software without manual intervention. Additionally, in 2012 a single particle tracking competition took place where different autonomous methods did better than other methods depending on the SNR, demonstrating the need for slight variation in approaches of tracking depending on differences in datasets [16].

While the false positive rate is not 0% and there is human bias, it is well documented that across fields research uses highly trained humans to track and classify objects as cells and also to manually validate automated tracking and classifying methods. The true positive rate over the false positive rate for the HELM classification method was reported to 0.92 [10].

These points have been incorporated into the revised manuscript as outlined below so the reader will understand the standards across the field and these caveats.

L196 –

Until the last few decades all microscopy-based cellular classification and tracking was done manually by trained researchers [51]. Now that computers have become capable of single particle tracking and classifying motion, many more particles may be tracked at a time in some datasets, but manual tracking remains the most accurate method for discriminating particles from noise and linking tracks of a single particle [52-57].

L230 –

A researcher manually reviewed the HELM classifications to confirm accuracy and, alongside this review process, identified morphology and optical property biosignatures.

In datasets where false positive and false negative identifications by HELM’s classifier were never below several dozen, moving particles were instead tracked using the Fiji plugin TrackMate, using LoG/DoG detection followed by Kalman or LAP tracking [53], or Manual Tracking.

L259 –

False positive rates for manual identification of biosignatures, while theoretically not zero, can be considered negligible [53, 54].

L286 –

Along with directional drift and displacement-vs.-time curves, HELM also uses other track parameters such as sinuosity and angle of displacement to identify motile particles. In lab monoculture samples, the true positive rate over the false positive rate for automated motile track detection was reported to be 0.92 [61]. Manual results remain superior to any automated results, however, largely due to poor stitching of automatic tracks. Manual tracking combined with plotting displacement curves in the case of slow-moving cells essentially eliminates false positive identification of motile tracks.

2. The small number of observed motile microbes and the absence of ground truth for some of the data sets means that there is no way to assess what the false positive rate is, and thus no way to verify the claims.

**Author response**: We agree that, ideally, we would have more robust datasets, but unfortunately, we cannot change the small number of usable recordings per sample and motile microbes that we have. The difficulties of getting to the sites and collecting good clean samples are non-trivial. With this type of remote work, when we recorded data, whether or not that data would be useable was not always clear until we got back to the lab and processed. Once recordings were analyzed a number of them were identified to be unusable due to misalignment of sample chamber, debris and sediment causing noise, or other unforeseen optical issues. We still believe in the importance of publishing the presence of these biosignatures and capabilities of the DHM throughout such a wide variety of environments. The shortcomings of these data are likely shortcomings that could be present on a future space exploration mission, and being able to interpret and determine what claims can be made are important.

While we are not able to give false positive or negative rates, we do believe that verification of data, including number of motile organisms, is very possible with other researchers following our analysis protocols. In our Image Processing section, we outline the protocol we used. Even though we do not use the same exact protocol for each data set, we believe this general protocol is robust and with some minor tweaking of parameters used would give reproducible results. As outlined in the above response we do not believe that a false positive rate is possible or essential to determine.

We have worked to include ground truthing for all the sites that we could. Below we have outlined the text where we have included the ground truthing data and added to the materials and methods section the methods used for ground truthing. Now the only site that does not have ground truthing is Mount Saint Helens glacial void. Even though the glacial void site does not have ground trothing, we still believe that this site should be included in our paper, because having a dataset with no ground truthing makes it closer to an extraterrestrial analog and gives the case for future research.

These points are being made in the manuscript by the following text:

L293 –

The number of observed motile organisms in our samples was highly variable; some sites had only a few organisms identified over many recordings while some had dozens per field of view. This variability led us to use a qualitative analytical approach where motile organisms were classified as either highly abundant (multiple motile cells in each frame), abundant (at least one motile cell per frame), present (motile cells in some but not all frames), or not present (no motile cells in any frame).

L374 –

7. Ground Truthing

Ground truthing included high resolution fluorescence imaging using dye staining and/or genetic sequencing. Both of these types of ground truthing data were collected for samples from seawater, sea ice brine, and cryopeg samples from **Utqiaġvik, AK. F**luorescence microscopy only was performed on the other seawater sites, sea ice brine, hot spring, and glacial melt samples. Gene sequencing data only was used to compare cell concentrations of desert and hyperalkaline spring systems. No ground truthing data were collected for ice from subglacial void in Mount Saint Helens. Having no ground truthing data to compare to DHM on a life detection mission is very possible and so we believe it is important to include results from Mount Saint Helens subglacial void ice samples where we have no ground truthing data. Additionally, Mount Saint Helens subglacial void is the first study of microbial biosignatures present in ice core samples of any glaciovolcanic environment, and observation of potential life there should inspire “return missions” to the site.

L729 –

This study highlights the importance of ground truthing to complement DHM data. Fluorescence imaging and genetic sequencing can provide crucial information to confirm imaging biosignatures.

3. Because the criteria for processing the images differ from recording to recording and depend on the intervention of an observer, it would be very difficult to replicate the results in future, independent studies.

**Author response**: We do not believe that this is true. There are only a handful of parameters that can be altered within our outlined protocol and after some training most researchers would have little issue identifying parameters that improve SNR, allowing them to identify tracks and classify motility.

4. A minor comment: the figures are overcompressed, and figure 3 in particular is unreadable in my copy of the manuscript.

**Author response**: Thank you for pointing this out. We have altered figure 3 to be more readable by increasing text and image size.

**Reviewer 4**

 (No Response)

**Author response**: Thank you for your approval for the publication of our manuscript.

References:

1.	Mullane KK, Nishiyama M, Kurihara T, Bartlett DH. Compounding deep sea physical impacts on marine microbial motility. Frontiers in Marine Science. 2023;10.

2.	Lindensmith CA, Rider S, Bedrossian M, Wallace JK, Serabyn E, Showalter GM, et al. A Submersible, Off-Axis Holographic Microscope for Detection of Microbial Motility and Morphology in Aqueous and Icy Environments. PLOS ONE. 2016;11(1):e0147700.

3.	Carpentier Solorio Y, Lemaître F, Jabbour B, Tastet O, Arbour N, Bou Assi E. Classification of T lymphocyte motility behaviors using a machine learning approach. PLOS Computational Biology. 2023;19(9):e1011449.

4.	Tinevez J-Y, Perry N, Schindelin J, Hoopes GM, Reynolds GD, Laplantine E, et al. TrackMate: An open and extensible platform for single-particle tracking. Methods. 2017;115:80-90.

5.	Bedrossian M, El-Kholy M, Neamati D, Nadeau J. A machine learning algorithm for identifying and tracking bacteria in three dimensions using Digital Holographic Microscopy. AIMS Biophysics. 2018;5(1):36-49.

6.	Riekeles M, Schirmack J, Schulze-Makuch D. Machine Learning Algorithms Applied to Identify Microbial Species by Their Motility. Life. 2021;11(1):44.

7.	Grossart H-P, Riemann L, Azam F. Bacterial motility in the sea and its ecological implications. Aquatic Microbial Ecology. 2001;25(3):247-58.

8.	Fenchel T. Eppur si muove: many water column bacteria are motile. Aquatic Microbial Ecology. 2001;24:197-201.

9.	Kæstel-Hansen J, De Sautu M, Saminathan A, Scanavachi G, Da Cunha Correia RFB, Nielsen AJ, et al. Deep learning assisted single particle tracking for automated correlation between diffusion and function. 2023.

10.	Wronkiewicz M, Lee J, Mandrake L, Lightholder J, Doran G, Mauceri S, et al. Onboard Science Instrument Autonomy for the Detection of Microscopy Biosignatures on the Ocean Worlds Life Surveyor. The Planetary Science Journal. 2024;5(1):19.

11.	Clarke DT, Martin-Fernandez ML. A Brief History of Single-Particle Tracking of the Epidermal Growth Factor Receptor. Methods and Protocols. 2019;2(1):12.

12.	Wilson RS, Yang L, Dun A, Smyth AM, Duncan RR, Rickman C, et al. Automated single particle detection and tracking for large microscopy datasets. Royal Society Open Science. 2016;3(5):160225.

13.	Kok RNU, Hebert L, Huelsz-Prince G, Goos YJ, Zheng X, Bozek K, et al. OrganoidTracker: Efficient cell tracking using machine learning and manual error correction. PLOS ONE. 2020;15(10):e0240802.

14.	Bruijning M, Visser MD, Hallmann CA, Jongejans E. trackdem: Automated particle tracking to obtain population counts and size distributions from videos in r. Methods in Ecology and Evolution. 2018;9(4):965-73.

15.	Huth J, Buchholz M, Kraus JM, Schmucker M, Von Wichert G, Krndija D, et al. Significantly improved precision of cell migration analysis in time-lapse video microscopy through use of a fully automated tracking system. BMC Cell Biology. 2010;11(1):24.

16.	Chenouard N, Smal I, De Chaumont F, Maška M, Sbalzarini IF, Gong Y, et al. Objective comparison of particle tracking methods. Nature Methods. 2014;11(3):281-9.
