# Supplementary material for: Extant life detection using label-free video microscopy in analog aquatic environments
Source: PLoS One. 2025 Mar 12;20(3):e0318239. doi: 10.1371/journal.pone.0318239 (PMC11902266; doi:10.1371/journal.pone.0318239)
Supplement: Attachment — Submitted filename: Response_to_reviewers_auresp_3.docx [file pone.0318239.s033.docx]

**PLOS ONE Manuscript PONE-D-23-22462**

**Response to Reviewers**

Dear PLOS ONE editor and reviewers,

Thank you for giving us the opportunity to submit a revised draft of the manuscript “**Extant Life Detection Using Label-Free Video Microscopy in Analog Aquatic Environments**” for publication in the PLOS ONE Journal. We recognize and greatly appreciate the time and labor that you all dedicated to providing input on improving our manuscript and are grateful for the insightful critiques on and valuable improvements to our paper. We have included many of the suggestions made. All changes within the manuscript are seen in red text. Please see below, in blue, for a point-by-point response to the reviewers’ comments and concerns. All page numbers refer to the revised manuscript file with tracked changes.

**Reviewers' Comments to the Authors and Respective Responses to Reviewers:**

**Reviewer #3:**

I apologize to the authors for the delay in getting them this review. The authors have made many changes in response to my and the other reviewers' comments. In response to my comments, they have now noted why they rely on manual identification. I think the revised justification is fine for the manuscript, which is trying to establish some baseline for future analyses. However, the authors note in Line 256 that "Trained experts manually identified and classified active motion, morphology, and optical properties as biosignatures through inspection of...". In the interests of reproducibility, it will be important for the authors to specify who the experts are and, more importantly, how they were trained. After the authors add this information, I would favor acceptance. I do not need to see the manuscript again.

**Author response**: Thank you for your thoughtful feedback and for taking the time to review our manuscript. We greatly appreciate your constructive suggestion to enhance the reproducibility of our work. We have outlined in the “Supporting Information” document specifically how researchers were trained to identify the biosignatures outlined in the paper and who did this analysis for our publication. This revised text is found below.

L205 –

**Biosignature Identification Training**

The experts involved in identifying and classifying active motion, morphology, and optical properties as biosignatures were individuals with advanced academic and professional backgrounds in relevant fields, e.g., microbiology, microscopy, biophysics, etc. The training process consisted of sessions that covered identification of active motion, morphology, and optical properties as biosignatures. These sessions included both theoretical instruction and practical exercises using multiple recordings of lab grown monocultures of different sized organisms and a subset of experimental data to ensure a consistent understanding of the classification criteria. To ensure high reliability, the experts underwent an initial calibration phase, during which they independently classified a set of samples, and their results were compared and discussed in group meetings. Discrepancies in classification were resolved through collaborative review and consensus-building, ensuring that the experts were aligned in their interpretation of the biosignatures. The final classification process was carried out under the established protocols outlined in the main text of this paper to ensure consistency across different experts. Biosignature identification and analysis of sites were performed by researchers Carl Snyder, Dr. Manuel Bedrossian, Dr. Casey Barr, and Dr. Jay Nadeau.

**Reviewer #4:**

Based on the earlier reviews and revisions, it is clear that there are some shortcomings in the statistics of the observed motion-based biosignatures (small number of observed motile microbes, absence of ground truthing, identifying motility manually instead of automated tracking for verification).

However, I think the authors have now very well explained 1) the exact purpose of this study, 2) the limits of getting good statistics in the extreme environments that were studied, 3) the need for such studies for future space missions, in which similar lack of statistics will likely be a reality.

In the Introduction of the paper (line 105-107) the authors write: “The hypothesis of this work is that while not every microbe will exhibit detectable motion, morphology, or optical property biosignatures, within most environments – no matter how extreme – some fraction of microbes will be identifiable by these biosignatures”. I think this is the important message, and makes this study relevant for future space missions.

Overall, the authors made every effort to provide ground truthing in the form of fluorescence microscopy and in some cases gene sequencing. They presented many DHM images, and videos. Applying the method to in situ samples in extreme environments, rather than ideal lab culture samples, is in my view a very important test. In that sense it forms a more realistic basis for future space missions.

So overall, although I recognize the shortcomings pointed out by the other reviewers (particularly with respect to statistical rigor), I think this study warrants publication. It is a very valuable basis for future studies that can add similar data and thereby increasing the statistics of this method.

**Author response**: Thank you for your kind words as well as taking time to review and suggest further edits. These comments help us strengthen our paper and made it easier to understand our work.
